# RSEM: accurate transcript quantification from RNA-Seq data with or without a reference genome

**DOI:** 10.1186/1471-2105-12-323

**Published:** 2011-08-04

**Authors:** Bo Li, Colin N Dewey

**Affiliations:** 1 Department of Computer Sciences, University of Wisconsin-Madison, Madison, WI, USA; 2 Department of Biostatistics and Medical Informatics, University of Wisconsin-Madison, Madison, WI, USA

## Abstract

**Background:**

RNA-Seq is revolutionizing the way transcript abundances are measured. A key challenge in transcript quantification from RNA-Seq data is the handling of reads that map to multiple genes or isoforms. This issue is particularly important for quantification with de novo transcriptome assemblies in the absence of sequenced genomes, as it is difficult to determine which transcripts are isoforms of the same gene. A second significant issue is the design of RNA-Seq experiments, in terms of the number of reads, read length, and whether reads come from one or both ends of cDNA fragments.

**Results:**

We present RSEM, an user-friendly software package for quantifying gene and isoform abundances from single-end or paired-end RNA-Seq data. RSEM outputs abundance estimates, 95% credibility intervals, and visualization files and can also simulate RNA-Seq data. In contrast to other existing tools, the software does not require a reference genome. Thus, in combination with a de novo transcriptome assembler, RSEM enables accurate transcript quantification for species without sequenced genomes. On simulated and real data sets, RSEM has superior or comparable performance to quantification methods that rely on a reference genome. Taking advantage of RSEM's ability to effectively use ambiguously-mapping reads, we show that accurate gene-level abundance estimates are best obtained with large numbers of short single-end reads. On the other hand, estimates of the relative frequencies of isoforms within single genes may be improved through the use of paired-end reads, depending on the number of possible splice forms for each gene.

**Conclusions:**

RSEM is an accurate and user-friendly software tool for quantifying transcript abundances from RNA-Seq data. As it does not rely on the existence of a reference genome, it is particularly useful for quantification with de novo transcriptome assemblies. In addition, RSEM has enabled valuable guidance for cost-efficient design of quantification experiments with RNA-Seq, which is currently relatively expensive.

## Background

RNA-Seq is a powerful technology for analyzing transcriptomes that is predicted to replace microarrays [1]. Leveraging recent advances in sequencing technology, RNA-Seq experiments produce millions of relatively short reads from the ends of cDNAs derived from fragments of sample RNA. The reads produced can be used for a number of transcriptome analyses, including transcript quantification [2-7], differential expression testing [8,9], reference-based gene annotation [6,10], and de novo transcript assembly [11,12]. In this paper we focus on the task of transcript quantification, which is the estimation of relative abundances, at both the gene and isoform levels. After sequencing, the quantification task typically involves two steps: (1) the mapping of reads to a reference genome or transcript set, and (2) the estimation of gene and isoform abundances based on the read mappings.

A major complication in quantification is the fact that RNA-Seq reads do not always map uniquely to a single gene or isoform. Previously, we have shown that properly taking read mapping uncertainty into account with a statistical model is critical for achieving the most accurate abundance estimates [7]. In this paper, we present a user-friendly software package, RSEM (RNA-Seq by Expectation Maximization), which implements our quantification method and provides extensions to our original model. A key feature unique to RSEM is the lack of the requirement of a reference genome. Instead, it only requires the user to provide a set of reference transcript sequences, such as one produced by a de novo transcriptome assembler [11,12]. Extensions to our original methodology include the modeling of paired-end (PE) and variable-length reads, fragment length distributions, and quality scores. In addition, a 95% credibility interval (CI) and posterior mean estimate (PME) are now computed for the abundance of each gene and isoform, along with a maximum likelihood (ML) estimate. Lastly, RSEM now enables visualization of its output through probabilistically-weighted read alignments and read depth plots.

Through experiments with simulated and real RNA-Seq data, we find that RSEM has superior or comparable quantification accuracy to other related methods. With additional experiments, we obtained two surprising results regarding the value of PE data and quality score information for estimating transcript abundances. Although a PE read provides more information than a single-end (SE) read, our experiments indicate that for the same sequencing throughput (in terms of the number of bases sequenced), short SE reads allow for the best quantification accuracy at the gene-level. And while one would assume that quality scores provide valuable information for the proper mapping of reads, we find that for RNA-Seq reads with Illumina-like error profiles, a model that takes into account quality scores does not significantly improve quantification accuracy over a model that only uses read sequences.

### Related work

A simple quantification method that was used in some initial RNA-Seq papers [13,14] and that is still used today is to count the number of reads that map uniquely to each gene, possibly correcting a gene's count by the "mappability" of its sequence [15] and its length. The major problems with this type of method are that it: (1) throws away data and produces biased estimates if "mappability" is not taken into account, (2) produces incorrect estimates for alternatively-spliced genes [16], and (3) does not extend well to the task of estimating isoform abundances. A couple of methods were later developed that addressed the first problem by "rescuing" reads that mapped to multiple genes ("multireads") [17,18]. Some other methods addressed the latter two problems, but not the first, by modeling RNA-Seq data at the isoform level [5]. Later, we developed the methodology behind RSEM, which addressed all of these issues by using a generative model of RNA-Seq reads and the EM algorithm to estimate abundances at both the isoform and gene levels [7]. Since the publication of the RSEM methodology, a number of methods utilizing similar statistical methods have been developed [3,4,6,19-22].

Of the methods developed, only RSEM and IsoEM are capable of fully handling reads that map ambiguously between both isoforms and genes, which the authors of both methods have shown is important for achieving the best estimation accuracies [4,7]. In contrast with IsoEM, RSEM is capable of modeling non-uniform sequence-independent read start position distributions (RSPDs), such as 3'-biased distributions that are produced by some RNA-Seq protocols [1]. In addition, RSEM can compute PME and 95% CIs, whereas IsoEM only produces ML estimates. Lastly, RSEM is the only statistical method that we are aware of that is designed to work without a whole genome sequence, which allows for RNA-Seq analysis of species for which only transcript sequences are available.

## Implementation

**Figure 1:**
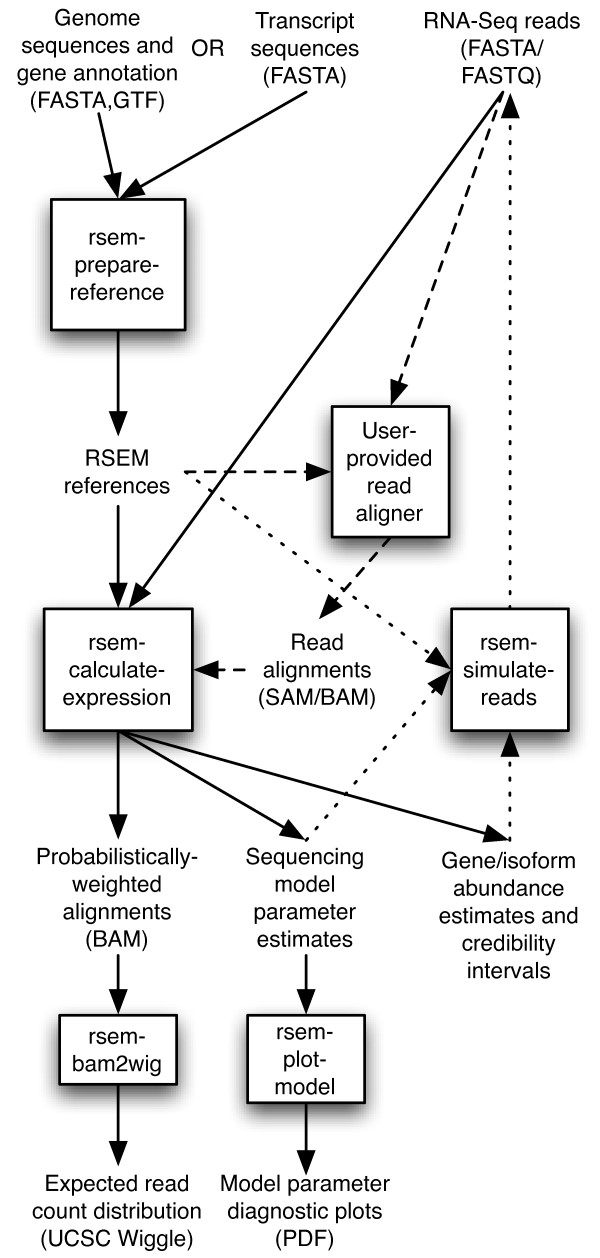
**The RSEM software workflow**. The standard RSEM workflow (indicated by the solid arrows) consists of running just two programs (rsem-prepare-reference and rsem-calculate-expression), which automate the use of Bowtie for read alignment. Workflows with an alternative alignment program additionally use the steps connected by the dashed arrows. Two additional programs, rsem-bam2wig and rsem-plot-model, allow for visualizing the output of RSEM. RNA-Seq data can also be simulated with RSEM via the workflow indicated by the dotted arrows.

A typical run of RSEM consists of just two steps. First, a set of reference transcript sequences are generated and preprocessed for use by later RSEM steps. Second, a set of RNA-Seq reads are aligned to the reference transcripts and the resulting alignments are used to estimate abundances and their credibility intervals. The two steps are carried out by the user-friendly scripts rsem-prepare-reference and rsem-calculate-expression. The steps of the RSEM workflow are diagrammed in Figure 1 and described in more detail in the following sections.

### Reference sequence preparation

RSEM is designed to work with reads aligned to transcript sequences, as opposed to whole genome sequences. There are several advantages to using transcript-level alignments. First, for eukaryotic samples, alignment of RNA-Seq reads to a genome is made complicated by splicing and polyadenylation. Reads that span splice junctions or that extend into poly(A) tails are challenging to align at the genome level, although there are tools available for aligning splice junction reads [23-25]. Second, using transcript-level alignments easily allows for analyses of samples from species without sequenced genomes but with a decently characterized transcriptome (perhaps via RNA-Seq transcriptome assembly [11,12]). Lastly, the total length of all possible transcripts is often much smaller than the length of the genome, allowing for faster alignment at the transcript-level.

A set of transcripts may be specified to rsem-prepare-reference in one of two ways. The simplest approach is to supply a FASTA-formatted file of transcript sequences. For example, such a file could be obtained from a reference genome database, a de novo transcriptome assembler, or an EST database. Alternatively, using the --gtf option, a gene annotation file (in GTF format) and the full genome sequence (in FASTA format) may be supplied. For commonly-studied species, these files may be easily downloaded from databases such as the UCSC Genome Browser Database [26] and Ensembl [27]. If the quality of existing gene annotations is in question, one can use a reference-based RNA-Seq transcriptome assembler, such as Cufflinks [28], to provide an improved set of gene predictions in GTF format. When gene-level abundance estimates are desired, an additional file specifying which transcripts are from the same gene may be specified (via the --transcript-to-gene-map option), or, if a GTF file is provided, the "gene_id" attribute of each transcript may be used to determine gene membership. With either method of specifying transcripts, RSEM generates its own set of preprocessed transcript sequences for use by later steps. For poly(A) mRNA analysis, RSEM will append poly(A) tail sequences to reference transcripts to allow for more accurate read alignment (disabled with --no-polyA). The scripts for preparing the reference sequences need only be run once per reference transcriptome as the transcript sequences are preprocessed in a sample-independent manner.

### Read mapping and abundance estimation

The rsem-calculate-expression script handles both the alignment of reads against reference transcript sequences and the calculation of relative abundances. By default, RSEM uses the Bowtie alignment program [29] to align reads, with parameters specifically chosen for RNA-Seq quantification. Alternatively, users may manually run a different alignment program and provide alignments in SAM format [30] to rsem-calculate-expression.

When using an alternative aligner, care must be taken to set the aligner parameters appropriately so that RSEM may provide the best abundance estimates. First, and most critically, aligners must be configured to report all valid alignments of a read, and not just a single "best" alignment. Second, we recommend that aligners be configured so that only matches and mismatches within a short prefix (a "seed") of each read be considered when determining valid alignments. For example, by default, RSEM runs Bowtie to find all alignments of a read with at most two mismatches in its first 25 bases. The idea is to allow RSEM to decide which alignments are most likely to be correct, rather than giving the aligner this responsibility. Since RSEM uses a more detailed model of the RNA-Seq read generation process than those used by read aligners, this results in more accurate estimation. Lastly, in order to reduce RSEM's running time and memory usage, it is useful to configure aligners to suppress the reporting of alignments for reads with a large number (e.g., > 200) of valid alignments.

While the original RSEM package only supported fixed-length SE RNA-Seq reads without quality score information, the new package supports a wide variety of input data types. RSEM now supports both SE and PE reads and reads of variable lengths. Reads may be given in either FASTA or FASTQ format. If reads are given in FASTQ format, RSEM will use quality score data as part of its statistical model. If quality scores are not provided, RSEM uses a position-dependent error model that we described previously [7].

After the alignment of reads, RSEM computes ML abundance estimates using the Expectation-Maximization (EM) algorithm for its statistical model (see Methods). A number of options are available to specify the model that is used by RSEM, which should be customized according to the RNA-Seq protocol that produced the input reads. For example, if a strand-specific protocol is used, the --strand-specific option should be specified. Otherwise, it is assumed that a read has equal probability of coming from the sense or antisense directions. The fragment length distribution is controlled by the --fragment-length- family of options, which are particularly important for SE analysis. For PE analysis, RSEM learns the fragment length distribution from the data. If the protocol produces read position distributions that are highly 5' or 3' biased, then the --estimate-rspd option should be specified so that RSEM can estimate a read start position distribution (RSPD), which may allow for more accurate abundance estimates [7].

In addition to computing ML abundance estimates, RSEM can also use a Bayesian version of its model to produce a PME and 95% CI for the abundance of each gene and isoform. These values are computed by Gibbs sampling (see Methods) and can be obtained by specifying the --calc-ci option. The 95% CIs are valuable for assessing differential expression across samples, particularly for repetitive genes or isoforms because the CIs capture uncertainty due to both random sampling effects and read mapping ambiguity. We recommend using the CIs in combination with the results of differential expression tools, which currently do not take into account variance from multiread allocation. The PME values may be used in lieu of the ML estimates as they are very similar, but have the convenient property of generally being contained within the 95% CIs, which is sometimes not the case for small ML estimates.

The primary output of RSEM consists of two files, one for isoform-level estimates, and the other for gene-level estimates. Abundance estimates are given in terms of two measures. The first is an estimate of the number of fragments that are derived from a given isoform or gene. We can only estimate this quantity because reads often do not map uniquely to a single transcript. This count is generally a non-integer value and is the expectation of the number of alignable and unfiltered fragments that are derived from a isoform or gene given the ML abundances. These (possibly rounded) counts may be used by a differential expression method such as edgeR [9] or DESeq [8]. The second measure of abundance is the estimated fraction of transcripts made up by a given isoform or gene. This measure can be used directly as a value between zero and one or can be multiplied by 10^6 ^to obtain a measure in terms of transcripts per million (TPM). The transcript fraction measure is preferred over the popular RPKM [18] and FPKM [6] measures because it is independent of the mean expressed transcript length and is thus more comparable across samples and species [7].

### Visualization

**Figure 2:**
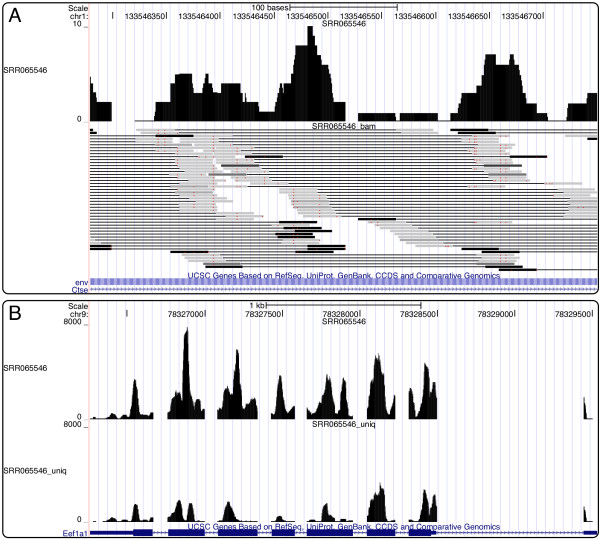
**RSEM visualizations in the UCSC Genome Browser**. Example visualizations of RSEM output from mouse RNA-Seq data set SRR065546 in the UCSC Genome Browser. (A) Simultaneous visualization of the wiggle output, which gives the expected read depth at each position in the genome, and the BAM output, which gives probabilistically-weighted read alignments. In the BAM track, paired reads are connected by a thin black line and the darkness of the read indicates the posterior probability of its alignment (black meaning high probability). (B) An example gene for which the expected read depth (top track) differs greatly from the read depth computed from uniquely-mapping reads only (bottom track).

RSEM can produce output for two different visualizations of RNA-Seq data as tracks in genome browsers, such as the UCSC Genome Browser [31]. When the --out-bam option is specified, RSEM maps read alignments from transcript to genomic coordinates and outputs the resulting alignments in BAM format [30]. Each alignment in the BAM file is weighted (using the MAPQ field) by the probability that it is the true alignment, given the ML parameters learned by RSEM. Visualization of the BAM file in a genome browser enables a user to see all of the read alignments and the posterior probabilities assigned to them by RSEM. The BAM file can be further processed by the rsem-bam2wig program to produce a UCSC WIG-formatted file that gives the expected number of reads overlapping each genomic position, given the ML parameters. Wiggle visualizations are useful for looking at the distributions of reads across transcripts. An example of the BAM and WIG visualizations within the UCSC Genome Browser is shown in Figure 2. To produce either visualization, one must have provided a GTF-formatted annotation file to the reference preparation script so that read alignments can be mapped back to genomic coordinates.

To help with diagnosing potential issues in RNA-Seq data generation or quantification, RSEM additionally allows for visualization of the sequencing model it learns from a given sample. This is accomplished by running the rsem-plot-model program on the output of rsem-calculate-expression. A number of plots are produced by rsem-plot-model, including the learned fragment and read length distributions, RSPD, and sequencing error parameters. Three of the plots generated for the RNA-Seq data set from SRA experiment SRX018974 [25] are shown in Additional file 1.

### Simulation

RSEM additionally allows for the simulation of RNA-Seq data sets according to the generative model on which it is based (see Methods). Simulation is performed by the rsem-simulate-reads program, which takes as input abundance estimates, sequencing model parameters, and reference transcripts (as prepared by rsem-prepare-reference). Typically, the abundance estimates and sequencing model are obtained by running RSEM on a real data set, but they may also be set manually.

## Results and Discussion

### Comparison to related tools

To evaluate RSEM, we compared its performance to a number of related quantification methods. We compared with IsoEM (v1.0.5) [4], Cufflinks (v1.0.1) [6], rQuant (v1.0) [2], and the original implementation of RSEM (v0.6) [32]. MISO [3], which uses a similar probabilistic model as RSEM, IsoEM, and Cufflinks, was not included in the comparison because it currently only computes the relative frequencies of alternative splice forms for each gene, not global transcript fractions. To make the comparisons fair, we ran Cufflinks only in its quantification mode. That is, it was configured to compute abundance estimates for the set of gene annotations that we provided to all methods and was not allowed to predict novel transcripts. Cufflinks and rQuant both require alignments of reads to a genome sequence and we used TopHat [24] for this purpose. TopHat was provided with the gene annotations and mean fragment length and was not allowed to predict novel splice junctions. For RSEM and IsoEM, which require alignments to transcript sequences, we used Bowtie [29]. As there are limited "gold-standard" data with which to evaluate the accuracy of RNA-Seq quantification methods, we tested the methods on both simulated and real data. On the simulated data, we additionally measured the computational performance (in terms of time and memory) of the methods.

#### Simulated data

As there are no published RNA-Seq data simulators, we performed experiments with the simulator included in the RSEM software package. This simulator uses the simple and widely-used model of RNA-Seq fragments being sampled uniformly and independently across all possible start sites from transcripts in a sample. The model used for the simulation is identical to that explicitly assumed by Cufflinks and IsoEM, and implicitly used by rQuant. Therefore, our simulation experiment is a test of how well the various methods perform when the data is generated from the model that they assume. We initially attempted to use an unpublished external simulation software package, Flux Simulator [33], but several bugs in the software prevented us from using it for the purposes of this paper.

We used the simulator to generate a set of 20 million RNA-Seq fragments in a non-strand-specific manner from the mouse transcriptome. Paired-end reads were simulated from these fragments, and a single-end read set was constructed by simply throwing out the second read of each pair. Two mouse reference transcript sets were used: the RefSeq annotation [34] and the Ensembl annotation [27] (see Methods). The RefSeq set is conservative with 20,852 genes and 1.2 isoforms per gene on average. In contrast, the Ensembl set has 22,329 genes and 3.4 isoforms per gene on average. We have made the simulation data for this experiment available on the RSEM website.

For each simulation set, we computed abundance estimates with the tested methods and measured the accuracy of the transcript fraction estimates using the median percent error (MPE), error fraction (EF), and false positive (FP) statistics that we used previously [7]. The MPE is the median of the percent errors of the estimated values from the true values. The 10% EF is the fraction of transcripts for which the percent error of the abundance estimate is greater than 10%. Lastly, the FP statistic is the fraction of transcripts with true abundance less than 1 TPM that are predicted to have abundance of at least 1 TPM. These statistics were calculated for three levels of estimates: (1) gene relative abundances, (2) global isoform relative abundances, and (3) within-gene isoform relative abundances.

**Figure 3:**
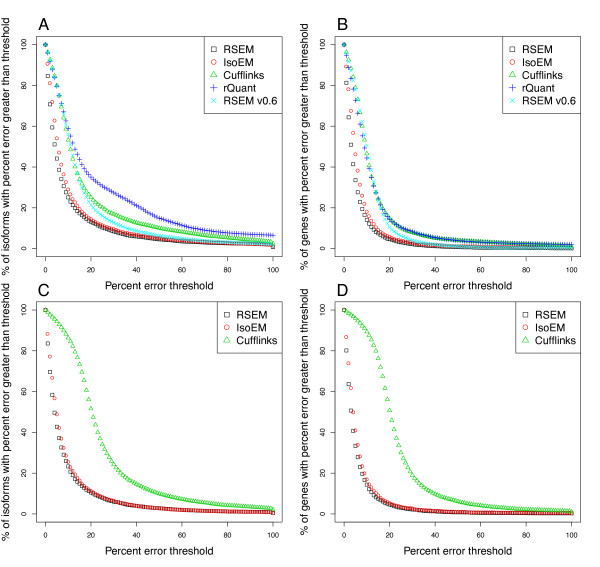
**Accuracy of four RNA-Seq quantification methods**. The percent error distributions of estimates from RSEM, IsoEM, Cufflinks, and rQuant on simulated RNA-Seq data. The error distributions of global isoform and gene estimates from PE data are shown in (A) and (B), respectively. Global isoform and gene estimate error distributions for SE data are shown in (C) and (D), respectively.

Figure 3 gives the distributions of the errors of the abundance estimates from the five methods on the RefSeq simulated sets, using a style of plot introduced by [4]. Table 1 gives the MPE, 10% EF, and FP rates for the methods. The results for the Ensembl simulated sets are shown in Additional file 2. RSEM v0.6 and rQuant were only run on the SE data, as they do not handle PE data.

**Table 1:** Accuracy measures for quantification methods applied to simulated data

	SE			PE		
Method	MPE	SE 10% EF	FP	MPE	PE 10% EF	FP
RSEM	**3.1**/**4.1**/**7.1**	**14.1**/**25.1**/**44.1**	**0.9**/**1.7**/**12.0**	**3.1**/**4.0**/5.2	**14.4**/**23.3**/35.7	**1.0**/1.8/11.3
IsoEM	4.6/5.6/8.0	18.1/29.0/45.4	1.7/3.2/31.3	4.0/4.8/**5.1**	16.9/25.5/**35.3**	1.2/**1.7**/**11.1**
Cufflinks	9.5/10.7/18.1	46.6/53.3/65.3	2.5/11.1/98.5	20.2/20.4/10.5	89.3/86.4/51.1	2.7/8.6/98.4
rQuant	8.9/12.6/43.9	44.4/58.9/89.0	7.0/16.9/99.1			
RSEM v0.6	10.1/11.2/10.4	50.5/56.0/50.7	1.4/2.9/39.5			

Accuracy of five RNA-Seq quantification methods on simulated SE and PE data. Values are given as gene/global isoform/within-gene isoform.

For both PE and SE reads, RSEM and IsoEM outperform Cufflinks and rQuant. There are likely two major reasons for the gap in performance between these two pairs of methods. First, Cufflinks and rQuant do not fully handle reads that map to multiple genes ("gene multireads"). Cufflinks uses a "rescue"-like strategy for an initial fractional allocation of multireads, which is roughly equivalent to one iteration of the EM algorithm used by RSEM and IsoEM. As for rQuant, it is not clear from [2] if and how this method handles gene multireads. A second reason for the performance gap is the fact that Cufflinks and rQuant require alignment of reads to the genome, not to a transcript set. As we discussed in the Implementation section, alignment of RNA-Seq reads to a genome sequence is challenging for eukaryotic species, whose RNA transcripts are spliced and polyadenylated.

The relative performance of the methods was similar across the RefSeq and Ensembl sets, although Cufflinks had surprisingly poor accuracy on the Ensembl set. A closer examination of the Cufflinks results revealed that this method was producing abnormally high abundance estimates on a subset of transcripts. This subset consisted of transcripts that were shorter (excluding poly(A) tails) than the mean fragment length (280 bases), indicating that the current implementation of Cufflinks does not properly handle short transcripts.

RSEM and IsoEM are comparable for PE data, but for SE data, RSEM is slightly more accurate. This relatively small improvement of RSEM over IsoEM is likely due to a more detailed implementation of poly(A) tail handling, which was not present in the original version of IsoEM and has only been recently introduced into its software. The improvement of the current version of RSEM over RSEM v0.6 is due to the modeling of fragment lengths for SE data, which was originally shown by [4] to improve accuracy.

#### MAQC data

It is challenging to benchmark RNA-Seq quantification methods on real data as we rarely know the "true" transcript abundances in a sample. Currently, qRT-PCR appears to be the most popular technology for producing "gold standard" abundance measurements, although without careful experimental design and data analysis it can give inaccurate results [35]. While RNA-Seq is generally accepted as being a more accurate quantification technology than microarrays [1], it remains to be seen whether it is also superior to qRT-PCR.

For our tests we used data generated from samples used in the Microarray Quality Control (MAQC) Project [36], as has been done in a number of other studies of RNA-Seq quantification accuracy [37,38]. The MAQC project evaluated a variety of microarray platforms and technologies, including TaqMan qRT-PCR, on two human RNA samples, one from brain tissue (HBR) and another from a mixture of tissue types (UHR). The TaqMan qRT-PCR measurements from this project consist of abundance values for a small subset (1,000) of genes, with four technical replicates on each of the two samples. Recently, three groups have generated RNA-Seq data on the two MAQC samples [25,37,39].

We applied the quantification methods on each of the MAQC RNA-Seq data sets and compared their abundance predictions to the qRT-PCR values. All methods were provided with the human RefSeq gene annotation. As for the simulation experiments, Cufflinks was only run in quantification mode and TopHat was only allowed to map to splice junctions present in the annotation. Cufflinks and IsoEM were run with and without their sequence-specific bias correction modes, which can improve quantification accuracy for RNA-Seq libraries generated with a random hexamer priming protocol, which was used for all of the MAQC RNA-Seq data. We did not run RSEM with its position-specific bias correction (RSPD) as this is only appropriate for oligo-dT primed RNA-Seq libraries, which generally have a bias towards reads originating from the 3' end of transcripts.

To assess the similarity of the RNA-Seq abundance predictions with the qRT-PCR measurements, we calculated the Pearson correlation of the logarithm of the abundance values. We used a log transformation to prevent the correlation values from being dominated by the most abundant transcripts. To avoid problems with zeros, correlation values were calculated for only those genes that were predicted to have non-zero abundance by qRT-PCR and all methods. We additionally computed the false positive (FP), true positive (TP), false negative (FN), and true negative (TN) counts for each method, where "positive" means non-zero predicted abundance and truth is determined by the qRT-PCR measurements.

The correlation values for the tested methods on each of the MAQC RNA-Seq samples are shown in Table 2. In general, the methods gave comparable correlation values for each sample. Confirming the results of [38], the bias correction mode of Cufflinks gave predictions with higher correlation than the other methods, particularly on the HBR samples. Unlike Cufflinks, the bias correction mode of IsoEM did not have a significant effect on its correlation with the qRT-PCR values for these samples. Spearman and Pearson correlation values computed without log-transformed abundances yielded similar results (Additional file 3). The TP, FP, TN, and FN counts for the methods were also comparable (Additional file 3).

**Table 2:** Correlation of quantification method predictions with MAQC qRT-PCR values

SRA ID	Read type	Sample	RSEM	IsoEM	IsoEM (C)	Cufflinks	Cufflinks (C)	rQuant
SRX016366	SE	HBR	0.69	0.68	0.68	0.71	**0.79**	0.72
SRX003926	SE	HBR	0.68	0.67	0.67	0.7	**0.73**	0.71
SRX018974	PE	HBR	0.69	0.69	0.69	0.69	**0.78**	NA
SRX016368	SE	UHR	0.71	0.71	0.72	0.72	**0.77**	0.72
SRX016369	SE	UHR	0.73	0.74	0.74	0.73	**0.76**	0.74
SRX016370	SE	UHR	0.74	0.75	0.75	0.74	**0.77**	0.75
SRX016371	SE	UHR	0.74	0.75	0.75	0.74	**0.77**	0.75
SRX016372	SE	UHR	0.75	0.75	0.75	0.74	**0.77**	0.76
SRX003927	SE	UHR	0.72	0.71	0.72	0.71	**0.74**	0.72

Correlation values (Pearson *r*^2 ^of log-transformed abundance values) were computed between the predictions of four methods and "gold-standard" values from qRT-PCR for nine different RNA-Seq data sets. IsoEM and Cufflinks were run with (C) and without their bias correction modes.

The lack of a clear distinction between the methods (except for Cufflinks with bias correction enabled) on these data sets can be explained by a number of factors. First, qRT-PCR measurements are only available for 1,000 (5%) out of a total of 19,005 genes in the RefSeq set. After filtering for the qRT-PCR genes that were consistent in their annotation with RefSeq and had non-zero abundance (see Methods), only 716 could be used for correlation analysis. Second, this set of genes is biased towards single-isoform genes and genes that have relatively unique sequences, reducing the ability of these data to distinguish those methods that are better at isoform quantification or multiread handling. The mean number of isoforms per gene in this set is 1.1, compared to 1.7 for all genes (*p *< 10^−115^, Mood's median test). Similarly, the mean "mappability" (see Methods) of genes in the set is 0.96, compared to 0.91 for all genes (*p *< 10^−6^). Lastly, biases in the qRT-PCR values, perhaps due to variable amplification efficiencies [35], may have resulted in an inaccurate gold standard.

#### Running time and memory

In addition to comparing the accuracies of the quantification methods, we also measured their running times and memory usage. For this purpose, we used our simulated mouse RefSeq data set of 20 million fragments, which is comparable in size to data produced by a single lane of the Illumina Genome Analyzer IIx. Table 3 lists the running times and peak memory usage for each method, on both SE and PE data. Additional file 4 gives the corresponding values for the simulated mouse Ensembl data set. All methods were run on an 8-core 2.93 GHz Linux server with 32 GB of RAM and hyper-threading enabled. Alignment with Bowtie against a transcript sequence set and quantification with RSEM uses the least amount of memory, at around 1.1 GB. The peak memory usage for Cufflinks and rQuant is due to running TopHat for aligning reads to the genome. The quantification programs for these two methods required 0.4 and 1.6 GB of memory, respectively, on the RefSeq data set. IsoEM is the fastest method, but has the largest memory requirement, up to 14 GB. It should be noted that the running times of the methods are not completely comparable, as RSEM and Cufflinks compute CIs in addition to ML estimates, whereas the other methods only compute ML estimates.

**Table 3:** Running time and memory usage of quantification methods on SE and PE data

	SE				PE			
Method	Alignment time	Quant. time	Total time	Peak Memory	Alignment time	Quant. time	Total time	Peak Memory
RSEM	24	50	74	1.1	15	50	66	1.1
IsoEM	5	6	12	12	14	10	24	14
Cufflinks	33	3	36	2.0	60	6	66	2.0
rQuant	33	183	216	2.0				
RSEM v0.6	9	22	31	1.1				

The alignment time, quantification time, total time, and peak memory usage of the tested quantification methods on SE and PE simulated data sets with 20 million fragments. Times are in minutes and memory is specified in GB.

The running time and memory required by RSEM scales linearly with the number of read alignments, which is generally proportional to the number of reads. Although the current version of RSEM has a parallelized EM algorithm, it is not faster than the original version for two reasons. First, the current version runs the EM algorithm for many more iterations to improve accuracy. On this data set, the current version ran for 4,802 iterations, compared to 643 for the older version. Second, the running time for the current version includes the time for computing 95% credibility intervals, which requires significant computation and was not a feature of the original version.

### Experimental results

With RSEM extended to model PE data and reads with quality score information, we set out to determine whether these more complex data types allow for improvement in abundance estimation accuracy. To this end, we performed two sets of simulation experiments. With the first set of experiments we compared the performance of PE reads against that of SE reads. With the second, we tested whether quality scores provide information that improves estimation accuracy.

#### Paired vs. single end reads

We previously showed that for SE RNA-Seq protocols, the number of reads is more important than the length of reads for increasing the accuracy of gene-level abundance estimates [7]. Given fixed sequencing throughput (in terms of the total number of bases), we found that the optimal read length was around 25 bases for SE RNA-Seq analysis in both mouse and maize. This result was confirmed by a later study [4]. Recent studies have reached the conclusion that PE reads can offer improved estimation accuracy over SE reads, particularly for isoforms of alternatively-spliced genes [3,4]. With RSEM now extended to model PE data, we decided to test these results with our own simulations.

We simulated RNA-Seq data with four different configurations: (1) 20 million, 35 base SE reads, (2) 20 million, 70 base SE reads, (3) 20 million, 35 base PE reads, and (4) 40 million 35 base SE reads. The latter three configurations give the same throughput in terms of the number of bases sequenced, and thus are the most comparable in terms of cost, given a simple economic model in which one pays per sequenced base. We simulated for both human and mouse, and with both RefSeq and Ensembl annotations, to determine if the species or annotation set is a factor. In addition to simulating with different species and annotation sets for each configuration, we also simulated with and without sequencing error to assess whether variable read alignment sensitivity had an impact.

Table 4 gives the MPE, 10% EF, and FP of the RSEM estimates computed from the RefSeq simulated data sets (Additional file 5 gives the corresponding values for the Ensembl sets). As expected, with the number of reads fixed, the 70 base reads gave better estimation accuracy than the 35 base reads. Confirming previous results [3,4], with the number of reads and total throughput fixed, PE reads improved estimation accuracy over SE reads (compare the PE accuracies with those of the SE 70 base accuracies). However, with the same sequencing throughput, short SE reads offered the highest estimation accuracy at the gene level. This result held across both species and regardless of whether reads contained sequencing errors. These results suggest that if the primary goal is the accurate estimation of gene abundances, then the sequencing of a large number of short SE reads is best. For example, given a choice between one Illumina lane of PE 35 base reads and two Illumina lanes of SE 35 base reads, our simulations show that the latter will provide the best overall quantification results for gene-level estimates. An additional advantage of using SE reads in this scenario is that two lanes of SE reads can be run in parallel whereas the two ends of a PE lane are currently generated one after the other. Thus, using short SE reads can save sequencing time. This result depends on the SE estimation procedure being provided with a fragment length distribution, as SE data is not easily used to automatically determine this distribution. However, this distribution can usually be obtained by other means ahead of time.

**Table 4:** Accuracies obtained from RNA-Seq data sets with various properties

Species	Seq. Error	Read type	Read length	Read number (×10^6^)	Throughput (MB)	MPE	10% EF	FP
M	N	SE	35	20	700	3.1/4.2/7.1	13.7/25.0/44.0	1.0/1.8/10.9
M	N	SE	70	20	1400	3.1/4.1/6.0	13.6/23.9/40.4	0.8/1.5/8.2
M	N	PE	35	20	1400	3.0/3.9/4.9	13.3/21.9/**34.2**	1.1/1.8/13.4
M	N	SE	35	40	1400	**2.3**/**3.1**/**4.8**	**8.4**/**18.5**/35.8	**0.7**/**1.3**/**10.5**

M	Y	SE	35	20	700	3.1/4.2/6.9	14.1/25.2/43.0	1.1/1.9/13.2
M	Y	SE	70	20	1400	3.0/4.0/6.0	14.2/24.4/40.6	1.1/1.6/9.5
M	Y	PE	35	20	1400	3.0/3.9/5.1	14.0/22.9/**35.5**	1.3/1.9/12.1
M	Y	SE	35	40	1400	**2.2**/**3.0**/**5.0**	**8.5**/**18.4**/35.7	**0.9**/**1.5**/**11.3**

H	N	SE	35	20	700	4.0/7.8/14.3	20.2/43.5/58.6	3.6/8.0/21.6
H	N	SE	70	20	1400	3.9/7.3/11.9	19.3/41.0/54.4	3.5/7.0/17.3
H	N	PE	35	20	1400	3.7/6.2/**9.0**	17.0/36.4/**47.3**	3.7/6.4/**14.0**
H	N	SE	35	40	1400	**2.9**/**5.7**/10.1	**13.4**/**35.4**/50.3	**2.7**/**6.3**/20.3

H	Y	SE	35	20	700	3.9/7.7/14.8	19.5/43.2/59.3	3.9/8.1/20.8
H	Y	SE	70	20	1400	3.8/7.2/12.4	19.2/40.8/55.2	3.9/7.1/17.7
H	Y	PE	35	20	1400	3.8/6.5/**9.2**	19.0/37.8/**48.2**	4.2/6.4/**13.9**
H	Y	SE	35	40	1400	**2.9**/**5.6**/10.3	**12.8**/**35.5**/50.8	**3.0**/**6.3**/18.8

Accuracy of abundance estimates from RNA-Seq data sets varying in species (H = human, M = mouse), sequencing error, type (SE or PE) of reads, number of reads, and length of reads. Values are given as gene/global isoform/within-gene isoform.

On the other hand, if the primary interest is in the relative frequencies of alternative splicing events within single genes, then PE data can provide more accurate estimates, depending on the transcript set. The result that the PE data show a larger accuracy improvement over SE data for the human RefSeq simulations is explained by the fact that the human RefSeq annotation has more isoforms per gene on average (1.6) than the mouse RefSeq annotation (1.2). This is further supported by the results of the simulations using the Ensembl annotations, which have significantly more isoforms per gene on average (6.3 for human and 3.4 for mouse). Thus, for species with genes that undergo a large number of alternative splicing events, PE data will likely be better for inferring the relative frequencies of these events. Although the results for gene-level and within-gene isoform-level estimates are clear, those for global isoform-level estimates are mixed. In some simulation sets, SE data performs better than PE data (with the same throughput), and in others, the opposite is true. This is explained by the fact that the global abundance of an isoform is the product of its gene's abundance and its within-gene abundance. Thus, one can improve global isoform abundance accuracy by producing better abundance estimates at either of the other two levels. Global isoform-level estimates are improved by SE data through more accurate gene-level estimates and by PE data through more accurate within-gene isoform estimates.

Overall, we suggest that researchers carefully consider the objectives of their RNA-Seq experiments before deciding on sequencing parameters, such as read length and number of reads. While one may be inclined to produce long and PE reads, it may be more cost efficient to use a larger number of SE reads if the only goal is quantification of gene abundances. If the goal is instead to analyze within-gene isoform frequencies or to perform non-quantification tasks such as transcriptome assembly, then PE reads should be preferred. To determine the optimal sequencing strategy for quantification with a particular transcript set, the RSEM simulation tool can be used.

#### The value of quality scores for RNA-Seq quantification

We performed simulation experiments to determine if the use of quality scores (rather than just the read sequences themselves) improves the accuracy of quantification with RNA-Seq data. Two SE simulations were performed, each with a different sequencing error model. The simulations used the mouse RefSeq transcript set as a reference. In the first simulation, an error was introduced at a given read position according to the theoretical probability of an error given the quality score at that position. That is, the probability that an error was introduced at a position with Phred quality score *q *was 10^−*q*/10^. In the second simulation, the probability of a sequencing error given a quality score *q *was determined from the training data (we call this the "empirical" model). For the two simulated data sets, we estimated abundances with RSEM using two different models: one that takes the quality scores into account (the "quality score" model), and a second that uses our original error model, which does not take into account quality scores and instead estimates a sequencing error model that is position and base-dependent (the "profile" model). The MPE, 10% EF, and FP statistics were calculated for the abundance estimates of the two RSEM models on the two simulated data sets (Table 5). We found that even when sequencing errors followed the theoretical probabilities given by the quality scores, the accuracy of the quality score model was practically indistinguishable from that of the profile model. Simulations with the Ensembl transcript set gave similar results (Additional file 6). This indicates that for the purposes of quantification from RNA-Seq data, quality scores from Illumina-generated reads provide little additional information. This does not suggest that sequencing errors do not need to be modeled, however. Instead, these results suggest that an effective sequencing error model can be learned from the read sequences alone. We stress that these results are only for the task of quantification. Applications such as SNP detection will certainly need to take quality score information into account.

**Table 5:** The effect of quality score modeling on quantification accuracy

Simulation model	Estimation model	MPE	10% EF	FP
theoretical	quality	3.1/4.1/7.2	13.8/25.2/43.5	1.0/1.8/11.6
theoretical	profile	3.1/4.1/7.2	13.9/25.3/43.6	1.0/1.8/11.7
empirical	quality	3.1/4.0/7.0	14.2/25.3/43.0	1.2/2.0/11.4
empirical	profile	3.1/4.1/7.0	14.3/25.4/43.2	1.1/2.0/11.2

Accuracy of abundance estimates from RNA-Seq data sets with different combinations of sequencing error models for simulation and estimation. Values are given as gene/global isoform/within-gene isoform.

## Conclusions

We have presented RSEM, a software package for performing gene and isoform level quantification from RNA-Seq data. Through simulations and evaluations with real data, we have shown that RSEM has superior or comparable performance to other quantification methods. Unlike other tools, RSEM does not require a reference genome and thus should be useful for quantification with de novo transcriptome assemblies. The software package has a number of other useful features for RNA-Seq researchers including visualization outputs and CI estimates. In addition, the software is user-friendly, typically requiring at most two commands to estimate abundances from raw RNA-Seq reads and uses reference transcript files in standard formats. Lastly, RSEM's simulation module is valuable for determining optimal sequencing strategies for quantification experiments. Taking advantage of this module, we have determined that a large number of short SE reads is best for gene-level quantification, while PE reads may improve within-gene isoform frequencies for the mouse and human transcript sets.

RSEM will continue to be developed to remain up to date with the latest sequencing technologies and research about details of the RNA-Seq protocol. Future work will include incorporating additional biases into the model, such as sequence-specific read position preferences [38,40] and transcript-specific read distributions [41]. We also intend to add support for color-space reads generated by ABI SOLiD sequencers and indels within read alignments.

## Availability and requirements

• **Project name: **RSEM

• **Project home page**: http://deweylab.biostat.wisc.edu/rsem

• **Operating systems: **Any POSIX-compatible platform (e.g., Linux, Mac OS X, Cygwin)

• **Programming languages: **C++, Perl

• **Other requirements: **Pthreads; Bowtie [29] for the default alignment mode of rsem-calculate-expression; R for rsem-plot-model.

• **License: **GNU GPL.

## Methods

### Statistical model

**Figure 4:**
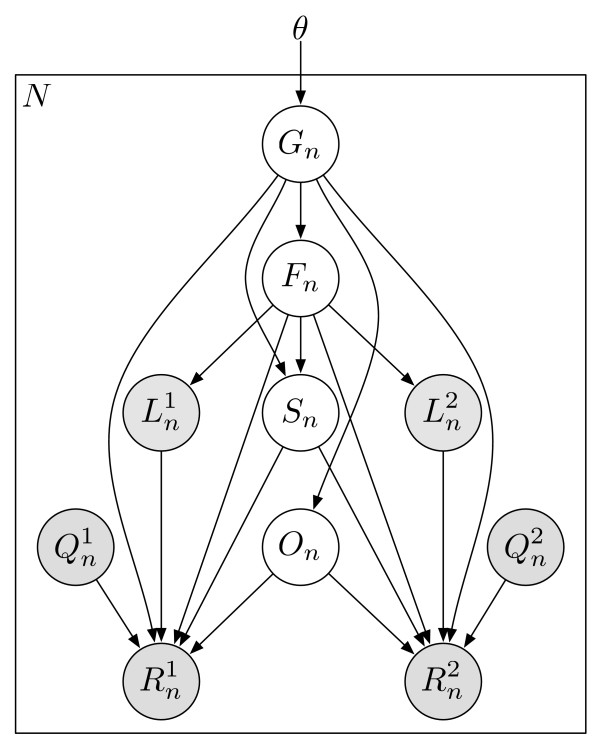
**The directed graphical model used by RSEM**. The model consists of *N *sets of random variables, one per sequenced RNA-Seq fragment. For fragment *n*, its parent transcript, length, start position, and orientation are represented by the latent variables *G_n_*, *F_n_*, *S_n _*and *O_n _*respectively. For PE data, the observed variables (shaded circles), are the read lengths ( and ), quality scores ( and ), and sequences ( and ). For SE data, , , and  are unobserved. The primary parameters of the model are given by the vector *θ*, which represents the prior probabilities of a fragment being derived from each transcript.

The statistical model used by RSEM can be represented by the directed graphical model shown in Figure 4. Compared to our original statistical model [7], this model has been extended in four ways. First, PE reads are now modeled, using a pair of observed random variables, *R*^1 ^and *R*^2^. For the case of SE reads, *R*^2 ^is treated as a latent random variable. Second, the length of the fragment from which a read or pair of reads is derived is now modeled and is represented by the latent random variable *F*. The distribution of *F *is specified using a global fragment length distribution *λ_F_*, which is truncated and normalized given that a fragment is derived from a specific transcript of finite length. That is,  where *ℓ_i _*is the length of transcript *i*. The use of a fragment length distribution for RNA-Seq quantification was first introduced by [6] for paired-end data and later described by [4] for single-end data.

A third extension allows the lengths of reads to vary (such as for 454 data). The length of a read is represented by the observed random variable *L *(or *L*^1 ^and *L*^2 ^for PE reads). Similar to the fragment length model, the distribution of *L *is specified using a global read length distribution *λ_R_*, which is truncated and normalized given a specific fragment length. In symbols, . Lastly, the quality scores for a read are now used to model the probability of that read's sequence. The quality score string for a read is represented by the random variable *Q*. For the purposes of quantification, we do not specify a distribution for the *Q *random variables, as they are observed and not dependent on any of the other random variables (i.e., we are only interested in the conditional likelihood of the reads given their quality scores). Rather than rely on the theoretical probabilities of errors implied by the quality scores, we use an empirical error function, *ε*. Given that read position *i *has quality score *q_i _*and is derived from the reference character *c*, the conditional probability of the read character *r_i _*is *P*(*r_i_|q_i_, c*) = *ε*(*r_i_, q_i_, c*). If quality scores are not available or reliable, then our position and reference character-dependent error model [7] may be used.

### Expectation-Maximation

Given a set of RNA-Seq data, RSEM's primary goal is to compute the ML values of the parameters, *θ*, of the model presented in the previous section, where *θ_i _*represents the probability that a fragment is derived from transcript *i *(with *θ*_0 _representing the "noise" transcript from which reads that have no alignments may be derived). Once estimated, the *θ *values are converted to transcript fractions (which we denote by *τ*) using the equation  where  is the effective length of transcript *i *[6], given by  for poly(A)- transcripts and  for poly(A)+ transcripts, where *ℓ_A _*is the length of a poly(A) tail. The effective length can be thought of as the mean number of positions from which a fragment may start within the sequence of transcript *i*.

RSEM computes approximate ML estimates for *θ *using the EM algorithm (for details, see [7]). The estimates are approximate because alignments are used to restrict the possible positions from which reads may be derived. During the first 20 iterations (and every 100 iterations) of EM, the parameters of the fragment length, RSPD, and sequencing error distributions are updated along with *θ*. During all other iterations, only the *θ *parameters are updated. This estimation strategy is an improvement over the original implementation of RSEM, which estimated all parameters other than *θ *before EM using uniquely-mapping reads. The algorithm is stopped when all *θ_i _*with value ≥ 10^−7 ^have a relative change of less than 10^−3^. After convergence, RSEM outputs the ML *τ *values, as well as the expected value of the number of RNA-Seq fragments derived from each transcript, given the ML parameters.

To speed up inference, reads with a large number (at least 200, by default) of alignments are filtered out. We additionally filter out reads that are likely to be derived from poly(A) tails, as aligners may not always detect that these reads have many alignments. Due to the alignment approximation and this filtering strategy, a straightforward application of the EM procedure described will lead to biased abundance estimates for transcripts that contain highly-repetitive sequences (including poly(A) tails). Therefore, we apply a slight modification to our ML estimator to adjust for this bias. For transcript *i*, we calculate a value *m_i_*, which is the probability that a read (fragment) generated from transcript *i *will not have a large number of alignments. In general, the value of *m_i _*depends on the fragment length distribution, the read length distribution, the RSPD, the strand-specificity of the protocol, and the length of a poly(A) tail. During the maximization step of EM, our modification is to set *θ_i _*to be proportional to *c_i_*/(*Nm_i_*), where *c_i _*is the expected number of fragments derived from transcript *i *and *N *is the total number of unfiltered fragments.

### Gibbs sampling

In addition to computing ML estimates, RSEM uses a Bayesian version of its model to compute PME and 95% CIs of abundances. In the Bayesian model, the *θ *parameters are treated as latent random variables with a Dirichlet prior distribution. The parameters of the Dirichlet distribution (*α*) are set to one, which makes the prior equivalent to a uniform distribution and the maximum a posteriori estimates of *θ *equal to the ML estimates.

RSEM computes PMEs and 95% CIs with a two-stage sampling process. First, a standard application of the collapsed Gibbs sampling algorithm [42] is used to obtain a sampled set of count vectors, where each vector represents the number of fragments that are mapped to each transcript. During each round of the Gibbs sampling algorithm, the true mapping of each fragment is resampled given the current mappings of all other fragments. The initial mapping of each fragment is sampled according to the ML parameters computed by the EM algorithm. The algorithm is run to sample 1000 count vectors.

The second stage of the sampling process involves sampling values of *θ *given each count vector sampled from the first stage. Given a count vector, *c*, a *θ *vector is sampled from its posterior distribution, which is simply a Dirichlet distribution with *α_i _*= *c_i _*+ 1. For each count vector, 50 *θ *vectors are sampled, resulting in 50,000 total samples for *θ*. The *θ *samples are converted to transcript fractions (*τ*) and then summarized to produce a PME and 95% CI for the abundance of each transcript.

To validate the CIs generated by RSEM, we simulated an RNA-Seq data set with the mouse RefSeq annotation and estimated CIs with RSEM from 50% credibility up to 95% credibility. We then computed the fraction of transcripts for which the true abundances fell within the credibility intervals, out of all transcripts with abundance at least 1 TPM (Table 6). The results indicate that the 95% credibility intervals are reasonably accurate and that these intervals are tight (since the fraction of correctly predicted transcript levels goes down in step with the credibility level). CIs estimated from data simulated with the mouse Ensembl annotation were less accurate (Additional file 7). We investigated why the CIs were less accurate on this set and found that many of the CIs were biased downward due to the Dirichlet prior and the larger number of transcripts in the Ensembl set. Although the CIs for the Ensembl set did not perform as well as those for the RefSeq set, we expect that they are still useful for comparing abundances across samples, as the biases in the CIs should be consistent. However, these results suggest that further work is needed to develop prior distributions that can better handle the large numbers of transcripts with zero abundance that are typical of RNA-Seq data sets.

**Table 6:** Accuracy of RSEM's credibility interval estimates

Credibility level	Isoforms with true abundance within estimated CI (%)	Genes with true abundance within estimated CI (%)
95	93.1	94.3
90	87.7	89.0
85	82.6	83.9
80	77.3	78.6
75	72.1	73.4
70	67.0	68.4
65	62.0	63.5
60	57.0	58.5
55	52.0	53.4
50	46.9	48.4

Accuracies of credibility intervals computed by RSEM for credibility levels ranging from 50% to 95%.

### Reference sequences

Two sources were used for reference transcript set annotations: the RefSeq gene annotations from the UCSC Genome Browser Database [26] and the Ensembl release 63 annotations [27]. The genome versions used for the RefSeq annotations of human and mouse were build 36.1 (UCSC hg18) and build 37 (UCSC mm9), respectively. For the Ensembl human annotation, build 37 (UCSC hg19) was used instead. Both the RefSeq and Ensembl annotations were filtered to remove non-coding genes and genes located on non-standard chromosomes (e.g., chr1_random and chr5_h2_hap1). In addition, we identified a small fraction of RefSeq genes that were located at multiple, non-overlapping positions and renamed them so that each gene originated from a unique locus.

### Simulation

The generative statistical model used by RSEM is easily used to simulate RNA-Seq data. In addition to the primary parameters of the model (e.g., abundances, fragment and read length distributions, and sequencing error model parameters), quality score information must be provided to simulate reads. For the purposes of the simulations in this paper, we used a first-order Markov chain model of quality scores to generate quality score strings for each read. The parameters of the simulation model were learned from real RNA-Seq data sets from the Sequence Read Archive (SRA). The mouse simulation parameters were learned from SRA accession SRX026632, which consists of ~ 4.2 million PE 35 base reads sequenced from a library of poly(A)+ RNA from C2C12 mouse myoblasts [3]. For the human simulations, we learned parameters from SRA accession SRX016368, which consists of ~ 93 million SE 35 base reads sequenced from a MAQC UHR sample [37]. As the human data were SE reads, RSEM was provided with a fragment length distribution with *μ *= 200 and *σ *= 29 in order to learn the other model parameters. However, for the simulations, both human and mouse data were generated with a fragment length distribution with *μ *= 280 and *σ *= 17, which was used in [3] for similar simulations. Lastly, to model the fact that the mRNAs have poly(A) tails, we appended 125 As to the end of each transcript.

### MAQC validation

TaqMan qRT-PCR measurements were downloaded from the Gene Expression Omnibus (GEO) (Platform GPL4097). For each sample, the abundance of a gene was taken as the mean of the values that passed the detection threshold for all probes assigned to the gene across all technical replicates. Following [37], a gene was considered expressed if 75% of its probes passed the detection threshold. The RefSeq transcript accessions listed for each gene in the GEO record were compared to the RefSeq accessions for each gene in the genome annotation. Only those genes for which the GEO accessions were a superset of the annotation accessions were kept. This was done to ensure that the RNA-Seq estimates were comparable to the values for the qRT-PCR probes, which are only guaranteed to correspond to the accessions given in the GEO record. This filtering resulted in a set of 716 genes, 656 and 618 of which were detected in UHR and HBR, respectively.

To analyze how representative the filtered qRT-PCR genes were of the entire human RefSeq gene set, we computed the "mappability" of each gene. For each isoform we generated all possible 35 base reads from its sequences and aligned them to the entire transcript set with Bowtie, allowing at most two mismatches. The mappability of an isoform was computed as the fraction of reads derived from it that only aligned with isoforms of its gene. The mappability of a gene was then computed as the mean of its isoform mappabilities.

## List of abbreviations

PE: paired-end; SE: single-end; ML: maximum likelihood; PME: posterior mean estimate; CI: credibility interval; MPE: median percent error; EF: error fraction; FP: false positive; RSPD: read start position distribution

## Authors' contributions

BL wrote the RSEM software, co-developed the methodology and experiments, carried out the computational experiments, and helped to draft the manuscript. CD co-developed the methodology and experiments, and wrote the manuscript. All authors read and approved the final manuscript.

## Supplementary Material

Additional file 1 **Example plots generated by rsem-plot-model**. Click here for file

Additional file 2 **Accuracy of four RNA-Seq quantification methods with the Ensembl reference set**. Click here for file

Additional file 3 **Additional accuracy measures for RNA-Seq predictions vs. qRT-PCR values for MAQC samples**. Click here for file

Additional file 4 **Running time and memory usage of quantification methods on SE and PE data with the Ensembl reference set**. Click here for file

Additional file 5 **Accuracies obtained from RNA-Seq data sets with various properties and the Ensembl reference set**. Click here for file

Additional file 6 **The effect of quality score modeling on quantification accuracy with the Ensembl reference set**. Click here for file

Additional file 7 **Accuracy of RSEM's credibility interval estimates with the Ensembl reference set**. Click here for file
