# Supplementary material for: RSEM: accurate transcript quantification from RNA-Seq data with or without a reference genome
Source: BMC Bioinformatics. 2011 Aug 4;12:323. doi: 10.1186/1471-2105-12-323 (PMC3163565; doi:10.1186/1471-2105-12-323)
Supplement: Additional file 1 — Example plots generated by rsem-plot-model. [file 1471-2105-12-323-S1.PDF]

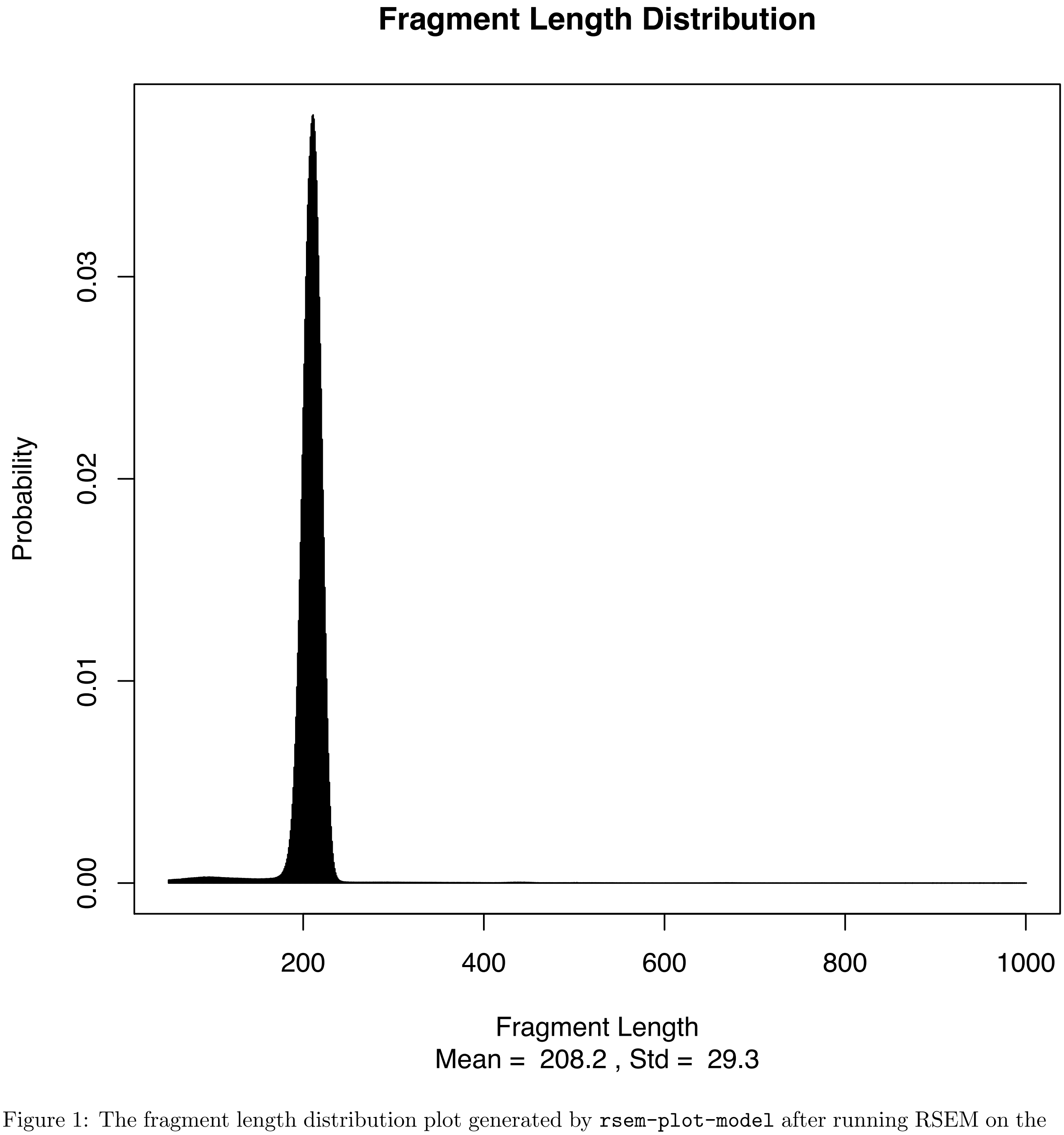

Figure 1: The fragment length distribution plot generated by `rsem-plot-model` after running RSEM on the RNA-Seq data from SRA experiment SRX018974.

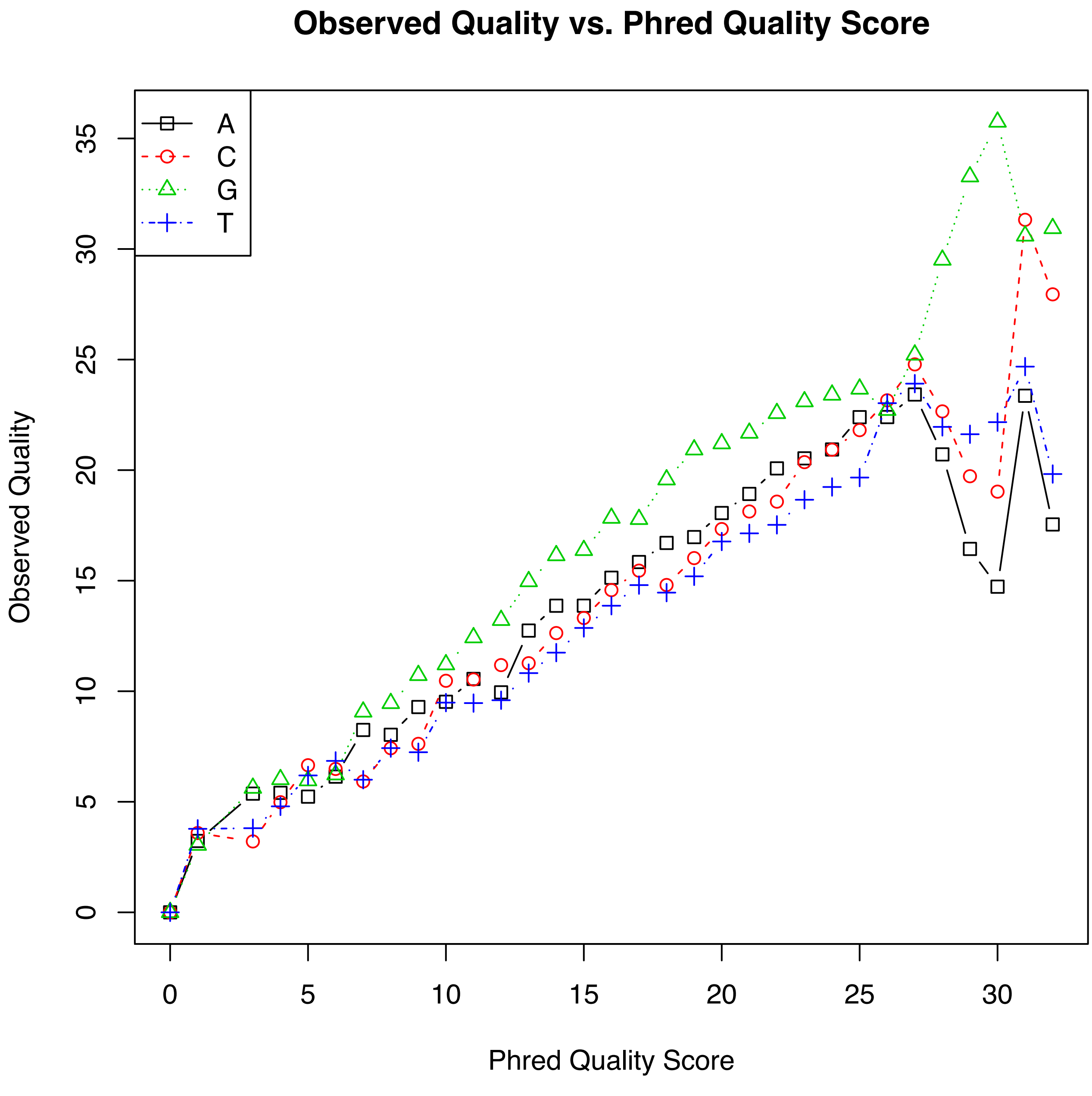

Figure 2: The sequencing error model summary plot generated by `rsem-plot-model` after running RSEM on the RNA-Seq data from SRA experiment SRX018974. The Phred quality scores are the scores produced from the sequencer and the “observed quality scores are estimates of the actual quality of the bases. On this data set, RSEM learns that the sequencer-produced quality scores are generally overestimates, although some of this difference can be attributed to the sample transcriptome having polymorphisms relative to the reference sequences.

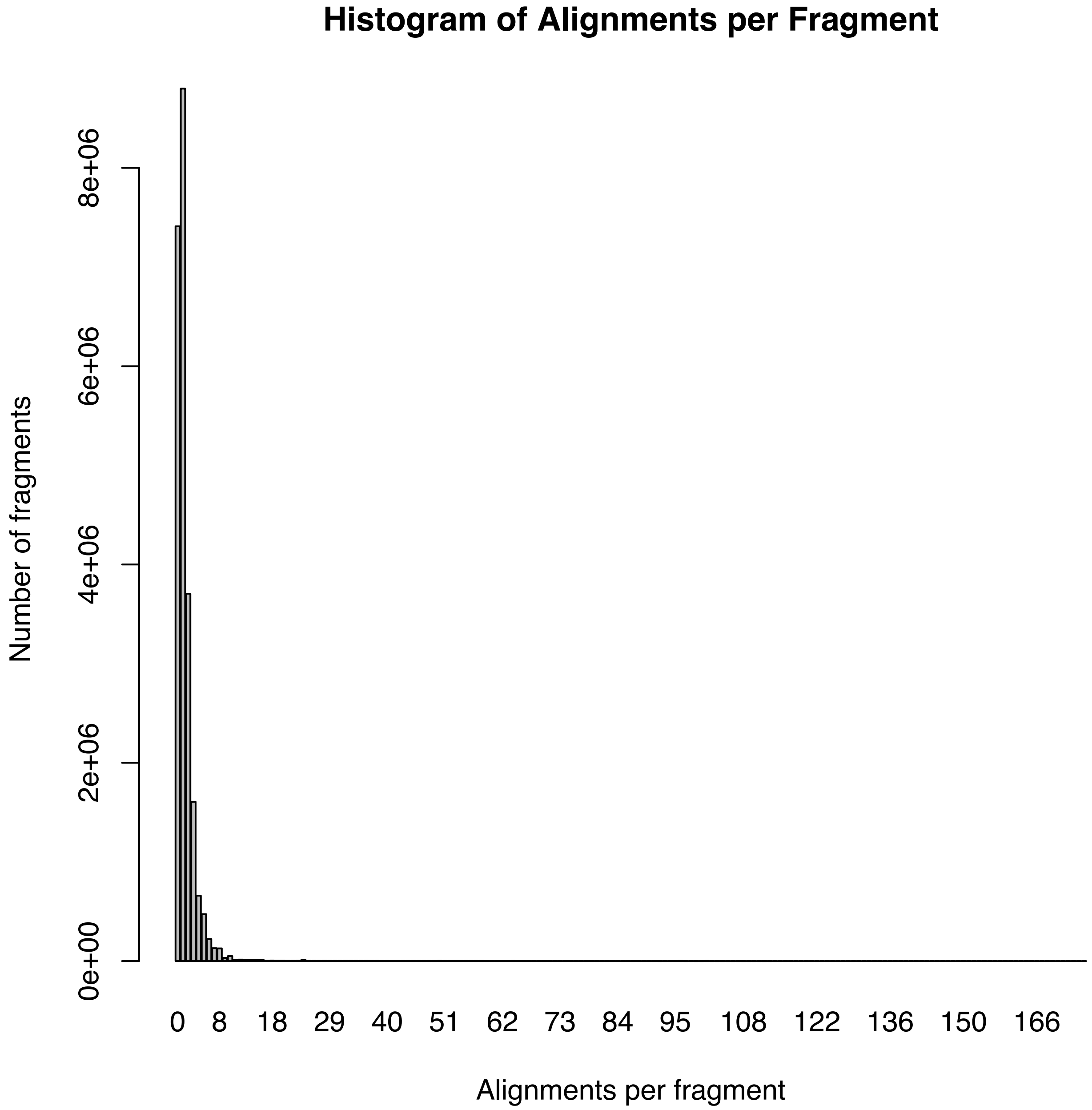

Figure 3: A diagnostic plot of the number of alignments per fragment generated by `rsem-plot-model` after running RSEM on the RNA-Seq data from SRA experiment SRX018974. This plot gives the user an idea of how common multireads are for a given data set.
