# Supplementary material for: RSEM: accurate transcript quantification from RNA-Seq data with or without a reference genome
Source: BMC Bioinformatics. 2011 Aug 4;12:323. doi: 10.1186/1471-2105-12-323 (PMC3163565; doi:10.1186/1471-2105-12-323)
Supplement: Additional file 2 — Accuracy of four RNA-Seq quantification methods with the Ensembl reference set. [file 1471-2105-12-323-S2.PDF]

Method	SE			PE		
	MPE	10% EF	FP	MPE	10% EF	FP
RSEM	**4.0/10.4/11.6**	**22.0/50.8/53.4**	**1.7/4.5/13.7**	**3.9/10.9**/11.7	**19.9/52.1**/53.7	2.0/4.9/13.8
IsoEM	6.1/12.3/13.0	27.7/56.2/56.0	2.7/4.9/17.4	5.2/11.2/**11.2**	23.6/53.2/**52.8**	**1.9/3.0/8.7**
Cufflinks	100.0/100.0/62.0	100.0/100.0/89.8	0.0/0.0/88.9	85.1/86.4/31.2	99.8/99.6/77.1	2.1/6.1/89.3
rQuant	14.7/51.4/47.6	64.6/89.2/88.3	7.3/30.4/90.9			
RSEM v0.6	11.2/18.2/16.8	56.0/72.0/62.2	2.2/5.4/18.6			

Table 1: Accuracy of five RNA-Seq quantification methods on simulated SE and PE data with the Ensembl mouse reference set. Values are given as gene/global isoform/within-gene isoform.

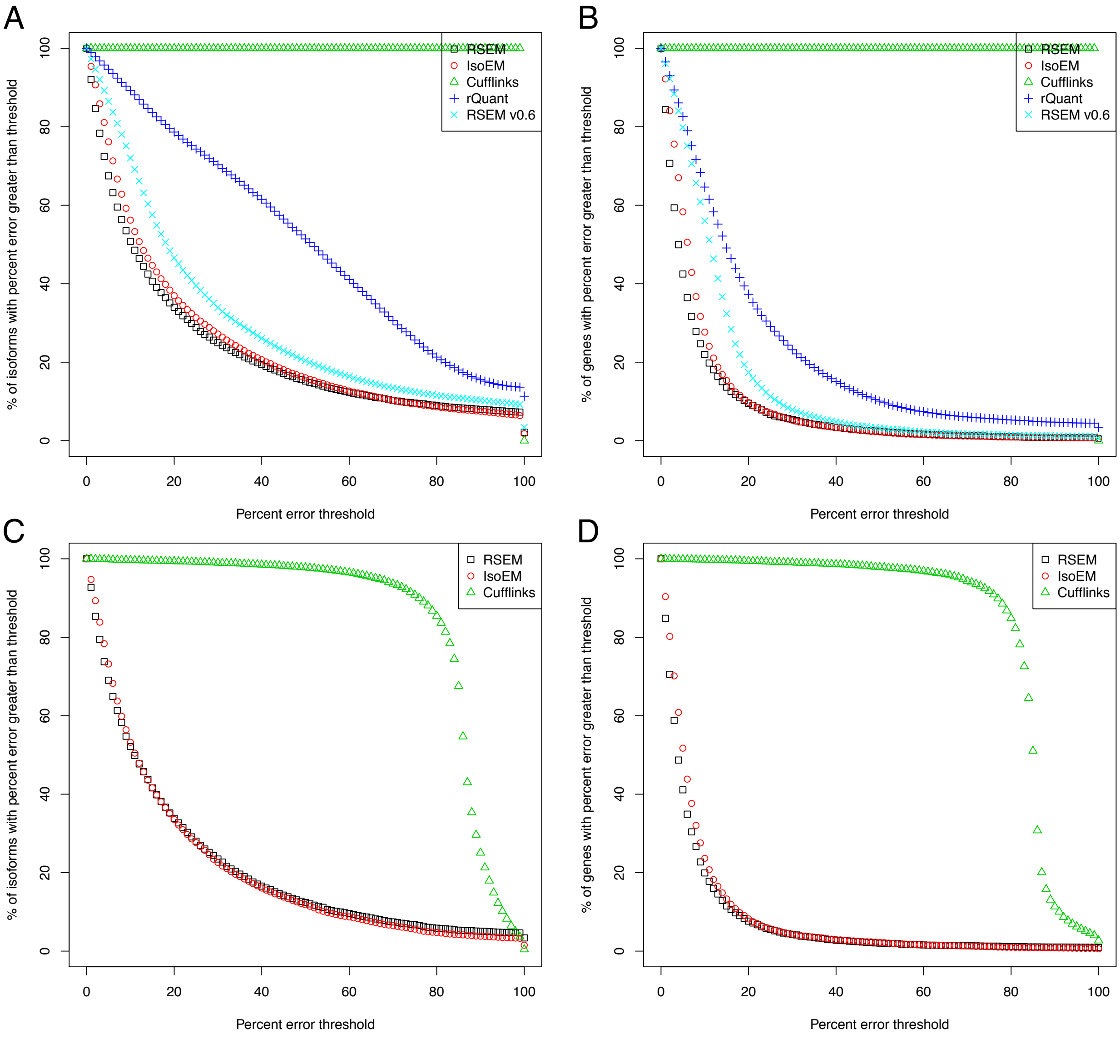

Figure 1: The percent error distributions of estimates from RSEM, IsoEM, Cufflinks, and rQuant on simulated RNA-Seq data with the Ensembl mouse reference set. The error distributions of global isoform and gene estimates from PE data are shown in (A) and (B), respectively. Global isoform and gene estimate error distributions for SE data are shown in (C) and (D), respectively.
