# Supplementary material for: RSEM: accurate transcript quantification from RNA-Seq data with or without a reference genome
Source: BMC Bioinformatics. 2011 Aug 4;12:323. doi: 10.1186/1471-2105-12-323 (PMC3163565; doi:10.1186/1471-2105-12-323)
Supplement: Additional file 3 — Additional accuracy measures for RNA-Seq predictions vs. qRT-PCR values for MAQC samples. [file 1471-2105-12-323-S3.PDF]

SRA ID	Read type	MAQC sample	RSEM	IsoEM	IsoEM (C)	Cufflinks	Cufflinks (C)	rQuant
SRX016366	SE	HBR	**0.73**	0.72	0.72	0.72	0.7	0.5
SRX003926	SE	HBR	0.65	0.66	0.68	0.62	**0.71**	0.61
SRX018974	PE	HBR	0.67	0.67	0.66	**0.7**	0.69	NA
SRX016368	SE	UHR	0.77	0.78	0.78	0.77	**0.83**	0.68
SRX016369	SE	UHR	0.82	0.83	0.83	0.81	**0.84**	0.75
SRX016370	SE	UHR	0.83	0.84	0.84	0.82	**0.85**	0.75
SRX016371	SE	UHR	0.82	0.83	0.83	0.81	**0.84**	0.76
SRX016372	SE	UHR	0.84	**0.85**	**0.85**	0.83	**0.85**	0.74
SRX003927	SE	UHR	0.75	0.77	0.8	0.71	**0.84**	0.75

Table 1: Pearson correlation (non-log-transformed values) of RNA-Seq predictions vs. qRT-PCR values for MAQC sample

SRA ID	Read type	MAQC sample	RSEM	IsoEM	IsoEM (C)	Cufflinks	Cufflinks (C)	rQuant
SRX016366	SE	HBR	0.66	0.66	0.66	0.66	**0.76**	0.66
SRX003926	SE	HBR	0.67	0.66	0.67	0.68	**0.7**	0.68
SRX018974	PE	HBR	0.64	0.64	0.64	0.64	**0.74**	NA
SRX016368	SE	UHR	0.72	0.72	0.72	0.72	**0.78**	0.72
SRX016369	SE	UHR	0.75	0.75	0.75	0.74	**0.78**	0.74
SRX016370	SE	UHR	0.76	0.76	0.75	0.75	**0.78**	0.75
SRX016371	SE	UHR	0.75	0.75	0.75	0.75	**0.78**	0.75
SRX016372	SE	UHR	0.76	0.76	0.75	0.75	**0.78**	0.75
SRX003927	SE	UHR	0.72	0.72	0.72	0.72	**0.75**	0.72

Table 2: Spearman correlation of RNA-Seq predictions vs. qRT-PCR values for MAQC samples

SRA ID	Read type	MAQC sample	RSEM	IsoEM	IsoEM (C)	Cufflinks	Cufflinks (C)	rQuant
SRX016366	SE	HBR	**52**	66	65	53	53	53
SRX003926	SE	HBR	32	40	39	**22**	**22**	**22**
SRX018974	PE	HBR	36	33	33	**27**	**27**	NA
SRX016368	SE	UHR	**36**	43	42	37	37	37
SRX016369	SE	UHR	29	38	38	**26**	**26**	**26**
SRX016370	SE	UHR	27	33	33	**24**	**24**	25
SRX016371	SE	UHR	26	36	35	**21**	**21**	22
SRX016372	SE	UHR	30	35	35	**27**	**27**	**27**
SRX003927	SE	UHR	**14**	20	20	**14**	**14**	**14**

Table 3: False positives of RNA-Seq predictions as determined by qRT-PCR values for MAQC samples

SRA ID	Read type	MAQC sample	RSEM	IsoEM	IsoEM (C)	Cufflinks	Cufflinks (C)	rQuant
SRX016366	SE	HBR	632	**636**	**636**	632	632	632
SRX003926	SE	HBR	602	**610**	608	598	598	599
SRX018974	PE	HBR	**614**	610	610	611	611	NA
SRX016368	SE	UHR	657	**662**	**662**	655	655	655
SRX016369	SE	UHR	645	**655**	**655**	645	645	646
SRX016370	SE	UHR	652	**659**	**659**	650	650	650
SRX016371	SE	UHR	649	**660**	**660**	651	651	652
SRX016372	SE	UHR	653	**656**	**656**	653	653	653
SRX003927	SE	UHR	622	**636**	634	617	617	617

Table 4: True positives of RNA-Seq predictions as determined by qRT-PCR values for MAQC samples

SRA ID	Read type	MAQC sample	RSEM	IsoEM	IsoEM (C)	Cufflinks	Cufflinks (C)	rQuant
SRX016366	SE	HBR	7	**3**	**3**	7	7	7
SRX003926	SE	HBR	37	**29**	31	41	41	40
SRX018974	PE	HBR	**25**	29	29	28	28	NA
SRX016368	SE	UHR	8	**3**	**3**	10	10	10
SRX016369	SE	UHR	20	**10**	**10**	20	20	19
SRX016370	SE	UHR	13	**6**	**6**	15	15	15
SRX016371	SE	UHR	16	**5**	**5**	14	14	13
SRX016372	SE	UHR	12	**9**	**9**	12	12	12
SRX003927	SE	UHR	43	**29**	31	48	48	48

Table 5: False negatives of RNA-Seq predictions as determined by qRT-PCR values for MAQC samples

SRA ID	Read type	MAQC sample	RSEM	IsoEM	IsoEM (C)	Cufflinks	Cufflinks (C)	rQuant
SRX016366	SE	HBR	**25**	11	12	24	24	24
SRX003926	SE	HBR	45	37	38	**55**	**55**	**55**
SRX018974	PE	HBR	41	44	44	**50**	**50**	NA
SRX016368	SE	UHR	**15**	8	9	14	14	14
SRX016369	SE	UHR	22	13	13	**25**	**25**	**25**
SRX016370	SE	UHR	24	18	18	**27**	**27**	26
SRX016371	SE	UHR	25	15	16	**30**	**30**	29
SRX016372	SE	UHR	21	16	16	**24**	**24**	**24**
SRX003927	SE	UHR	**37**	31	31	**37**	**37**	**37**

Table 6: True negatives of RNA-Seq predictions as determined by qRT-PCR values for MAQC samples
