# Supplementary material for: RSEM: accurate transcript quantification from RNA-Seq data with or without a reference genome
Source: BMC Bioinformatics. 2011 Aug 4;12:323. doi: 10.1186/1471-2105-12-323 (PMC3163565; doi:10.1186/1471-2105-12-323)
Supplement: Additional file 4 — Running time and memory usage of quantification methods on SE and PE data with the Ensembl reference set. [file 1471-2105-12-323-S4.PDF]

Method	SE				PE			
	Alignment time	Quant. time	Total time	Peak Memory	Alignment time	Quant. time	Total time	Peak Memory
RSEM	15	140	155	1.2G	22	120	142	1.3G
IsoEM	7	17	24	14G	18	24	42	15G
Cufflinks	28	3	31	2G	51	6	57	2G
rQuant	28	452	480	2G				
RSEM v0.6	31	52	83	2.1G				

Table 1: The alignment time, quantification time, total time, and peak memory usage of the tested quantification methods on SE and PE simulated data sets with the Ensembl mouse annotations and 20 million fragments. Times are in minutes and memory is specified in GB.
