# Supplementary material for: RSEM: accurate transcript quantification from RNA-Seq data with or without a reference genome
Source: BMC Bioinformatics. 2011 Aug 4;12:323. doi: 10.1186/1471-2105-12-323 (PMC3163565; doi:10.1186/1471-2105-12-323)
Supplement: Additional file 5 — Accuracies obtained from RNA-Seq data sets with various properties and the Ensembl reference set. [file 1471-2105-12-323-S5.PDF]

Species	Seq. Error	Read type	Read length	Read number ($\times 10^6$)	Throughput (MB)	MPE	10% EF	FP
M	N	SE	35	20	700	4.0/10.1/11.5	21.6/50.3/53.3	1.4/4.4/13.3
M	N	SE	70	20	1400	3.8/9.5/10.4	20.5/48.7/51.0	1.3/3.4/10.8
M	N	PE	35	20	1400	3.5/8.1/**7.5**	16.7/44.4/**43.2**	**0.9**/**1.5**/**4.3**
M	N	SE	35	40	1400	**2.8**/**7.2**/8.0	**14.2**/**42.1**/44.6	1.1/3.1/12.5
M	Y	SE	35	20	700	4.0/10.3/11.4	21.4/50.7/53.3	1.6/4.4/13.5
M	Y	SE	70	20	1400	3.8/9.6/10.3	20.1/48.8/50.6	1.5/3.5/10.8
M	Y	PE	35	20	1400	3.4/8.2/**7.7**	16.5/44.0/**43.6**	1.5/**1.8**/**5.0**
M	Y	SE	35	40	1400	**2.8**/**7.5**/8.2	**14.3**/**42.8**/45.1	**1.1**/3.3/12.5
H	N	SE	35	20	700	5.9/19.1/20.3	33.3/67.1/69.0	5.4/9.7/21.3
H	N	SE	70	20	1400	5.7/17.5/18.2	31.7/65.4/66.8	5.2/8.7/18.6
H	N	PE	35	20	1400	4.6/**12.6**/**12.4**	24.8/**57.0**/**56.7**	4.2/**5.1**/**9.7**
H	N	SE	35	40	1400	**4.4**/14.1/14.9	**24.6**/59.5/61.4	**3.9**/7.7/21.5
H	Y	SE	35	20	700	6.0/19.2/20.4	33.4/67.3/69.1	6.3/10.0/21.4
H	Y	SE	70	20	1400	5.8/17.4/18.3	32.1/65.0/66.4	5.8/8.9/18.7
H	Y	PE	35	20	1400	4.8/**13.0**/**12.8**	26.4/**57.7**/**57.6**	4.9/**5.7**/**10.4**
H	Y	SE	35	40	1400	**4.4**/13.8/14.5	**24.5**/58.9/60.7	**4.8**/7.8/21.4

Table 1: Accuracy of abundance estimates from RNA-Seq data sets simulated with Ensembl annotations and varying in species (H=human, M=mouse), sequencing error, type (SE or PE) of reads, number of reads, and length of reads. Values are given as gene/global isoform/within-gene isoform.
