# Supplementary material for: RSEM: accurate transcript quantification from RNA-Seq data with or without a reference genome
Source: BMC Bioinformatics. 2011 Aug 4;12:323. doi: 10.1186/1471-2105-12-323 (PMC3163565; doi:10.1186/1471-2105-12-323)
Supplement: Additional file 6 — The effect of quality score modeling on quantification accuracy with the Ensembl reference set. [file 1471-2105-12-323-S6.PDF]

Simulation model	Estimation model	MPE	10% EF	FP
theoretical	quality	4.0/10.4/11.8	21.9/51.0/53.8	1.7/4.5/13.6
theoretical	profile	4.1/10.5/11.8	21.9/51.0/53.8	1.8/4.5/13.8
empirical	quality	4.0/10.2/11.6	21.7/50.5/53.2	1.6/4.5/13.7
empirical	profile	4.0/10.2/11.6	21.7/50.5/53.4	1.6/4.6/13.7

Table 1: Accuracy of abundance estimates from RNA-Seq data sets simulated with Ensembl annotations and with different combinations of sequencing error models for simulation and estimation. Values are given as gene/global isoform/within-gene isoform.
