# Supplementary material for: RSEM: accurate transcript quantification from RNA-Seq data with or without a reference genome
Source: BMC Bioinformatics. 2011 Aug 4;12:323. doi: 10.1186/1471-2105-12-323 (PMC3163565; doi:10.1186/1471-2105-12-323)
Supplement: Additional file 7 — Accuracy of RSEM's credibility interval estimates with the Ensembl reference set. [file 1471-2105-12-323-S7.PDF]

Credibility level	Isoforms with true abundance within estimated CI (%)	Genes with true abundance within estimated CI (%)
95	75.7	80.6
90	68.6	73.1
85	62.7	67.3
80	57.5	61.9
75	53.1	57.1
70	48.5	52.4
65	44.4	48.3
60	40.6	44.0
55	36.6	40.2
50	33.0	36.4

Table 1: Accuracies of credibility intervals computed by RSEM on mouse Ensembl simulated data for credibility levels ranging from 50% to 95%.
